# Genomic High Plains Wheat Mosaic Virus Sequences from Australia: Their Phylogenetics and Evidence for Emaravirus Recombination and Reassortment

**DOI:** 10.3390/v15020401

**Published:** 2023-01-31

**Authors:** Roger A. C. Jones, Ines Vazquez-Iglesias, Sam McGreig, Adrian Fox, Adrian J. Gibbs

**Affiliations:** 1 UWA Institute of Agriculture, University of Western Australia, Crawley, WA 6009, Australia; 2 Department of Primary Industries and Regional Development, South Perth, WA 6151, Australia; 3 Fera Science Ltd., York Biotech Campus, York YO41 1LZ, UK; 4 Emeritus Faculty, Australian National University, Canberra, ACT 2600, Australia

**Keywords:** wheat, virus disease, High Plains wheat mosaic virus, Australia, high-throughput sequencing, phylogenetics, recombination, reassortment, biosecurity

## Abstract

High Plains wheat mosaic virus (HPWMoV) causes a serious disease in major wheat-growing regions worldwide. We report here the complete or partial genomic sequences of five HPWMoV isolates from Australian wheat samples. Phylogenetic analysis of the nucleotide sequences of the eight genomic segments of these five isolates together with others from Genbank found all eight genes formed two lineages, L1 and L2. L1 contained a single isolate from Colorado in the North American Great Plains Region (GPR), and L2 had two unresolved clusters, A and B, of isolates from Australia and the GPR. A quarter of the L2B isolate sequences of the nucleocapsid gene (RNA3) were recombinant, which is unexpected as little evidence of recombination exists in viruses with negative single-stranded RNA genomes. Phylogenies calculated from the amino acid sequences of HPWMoV’s RNA-dependent RNA-polymerase (RNA1), glycoprotein (RNA2), and nucleocapsid protein (RNA3) showed they were closest to those of Palo Verde broom virus. However, its movement protein (RNA4) was closer to those of Ti ringspot-associated and common oak ringspot-associated viruses, indicating the RNA4 segments of their ancestors reassorted to produce the current emaraviruses. To avoid increased yield losses from co-infection, biosecurity measures are advised to avoid HPWMoV introduction to countries where wheat streak mosaic virus already occurs.

## 1. Introduction

Wheat occupies more land than any other staple food crop worldwide and is the second most important global food crop after maize (corn) [1]. Many harmful virus diseases afflict wheat [2], and one of the most destructive is wheat streak mosaic disease (WSMD). WSMD’s most characteristic symptoms are plant stunting and a streaky yellow leaf mosaic. It is associated with three viruses that all share *Aceria tosichella* (WCM, the wheat curl mite) as their vector: wheat streak mosaic virus (WSMV; genus *Tritimovirus*, family *Potyviridae*), Triticum mosaic virus (TriMV; genus *Poacevirus*, family *Potyviridae*), and High Plains wheat mosaic virus (HPWMoV; genus *Emaravirus*, family *Fimoviridae*). To comply with the latest International Committee for Virus Nomenclature recommendations [3], in 2021, the species HPWMoV was given the binomial virus name *Emaravirus tritici*. WSMD causes the greatest damage to wheat crops when two or more of its causal viruses infect the same plant, which often occurs in wheat crops in the North American Great Plains Region (GPR). This co-infection is frequent because WCM transmits all three wheat viruses, and both their disease cycles and their epidemiology are greatly influenced by its ecology and behavior [4,5,6,7,8,9,10,11,12,13]. WSMV is present on all continents apart from Antarctica and is the cause of a major global wheat epidemic [10,14,15,16]. TriMV has not yet been reported outside of North America. HPWMoV (synonyms: High Plains virus, wheat mosaic virus, and maize red stripe virus) not only causes significant disease in the USA’s GPR [6,17,18], but also infects wheat crops in Canada’s GPR [19], Ukraine in Europe [12,20], Argentina in South America [21], and Australia [15,22,23,24]. It is transmitted with low efficiency from seed to seedlings of sweet corn [18,25,26], which is a high-sugar variant of maize. Although its seed transmission in wheat is suspected, but has not yet been confirmed, such transmission through the international wheat trade would explain its increasing global distribution.

The optimum temperature range is 24–27 °C for WCM population increase, as this tiny eriophyid mite vector species requires only 10 days to produce a new generation at these temperatures. Therefore, as climate change advances, global warming is projected to magnify the major global WSMV epidemic in many of the world’s wheat-growing regions by enhancing its spread [27,28,29,30]. The same would also be expected to apply to the other WCM-transmitted wheat viruses that cause WSMD (HPWMoV, TriMV) in regions in which they occur.

HPWMoV has an octapartite, negative-sense RNA genome that, in its virions, is coated with thread-like molecules of a 32 kDa nucleocapsid (NC), and these complexes are contained inside 80–200 nm diameter virions that have double membrane envelopes. Its genome’s eight single-stranded RNA components each have a single open reading frame (ORF) encoding a single protein [18,31,32,33,34,35,36]. Tatineni et al. [35] reported that in phylogenies of the amino acid (aa) sequences of three HPWMoV proteins from North American isolate NE [RNA-dependent RNA polymerase (RdRp), glycoprotein precursor protein, and NC], amongst other emaraviruses, these were closest to those of raspberry leaf blotch virus (RLBV). These three proteins were encoded by HPWMoV’s RNA1 (RdRp), RNA2 (GP), or RNA3 (NC), and those encoded by its RNA4-RNA8 were P4-P8. Its P4-P6 proteins resembled those of other emaraviruses, but the proteins encoded by its RNA7 and RNA8 lacked sequence homology with any other proteins present in Genbank. There were two distinct RNA3 aa sequences (3A and 3B), but only one aa sequence was present for each of the other seven RNAs [35].

When Stewart [37] compared the NC protein aa sequences of HPWMoV isolates NE, KS04, ABC58222, and TX96 [33,35,38] with those of six further North American HPWMoV isolates (H1, K1, W1, KS7, and CGI), they split into two main lineages (L1 and L2), one of which (L2) comprised two clusters, A and B. Three isolates (GG1, KS7, and NE) had two RNA3 sequences (3A and 3B), one of which fit into each cluster. Why some isolates have one and others have two RNA3 aa sequences is unknown [18]. The partial NC nucleotide (nt) sequences of three Ukrainian HPWMoV isolates formed a separate subcluster within cluster L2A, whereas those of two Australian (KT013206 and KC33741-2) and five Argentinian isolates were within the same L2B subcluster [12].

In Australia, HPWMoV was first detected in 2003 in preserved leaf samples from wheat-growing regions of New South Wales (NSW), the Australian Capital Territory, Queensland, South Australia, and Victoria [22,39]. During 2006–2011, it was detected again in preserved leaf samples collected in the NSW grain belt, occurring mainly in mixed infections with WSMV [24]. A partial sequence of an HPWMoV isolate from NSW was obtained (KT013206). In 2012, HPWMoV was detected in wheat leaf samples from commercial crops and a field experiment in Western Australia (WA) [23]. Two of the HPWMoV isolates obtained had nt sequences (KC33741-2) that were identical (100% nt identity) to the RNA3B sequence of the Nebraska isolate (NE), which itself has 100% nt identities with the RNA3B sequences of isolates KS7, CG1, and RA02 from the neighboring Kansas state. This indicated HPWMoV might have been introduced to Australia in infected maize or wheat seed from the USA’s central GPR [23]. Here, we report a phylogenetic study comparing the genomic sequences of five Australian HPWMoV isolates with those of HPWMoV currently available from other world regions, together with an assessment of the phylogenetic relatedness of HPWMoV to other emaraviruses.

## 2. Materials and Methods

### 2.1. Sample Origins and Sequencing

A recent study that included silica gel-preserved survey samples BCWS5, BCHPV1, HP1G, and HP2W from south-west Australia found WSMV was present in each of them [16]. Although HPWMoV was also present, this part of the study was excluded and is now reported here. The finding of HPWMoV alone in sample BCHPV2, which came directly from an infected leaf of the culture host sweet corn cv. Snow Gold provided by Brenda Coutts, is also reported here. Table 1 provides details of sample names, viruses present, isolation years, survey sample collection sites, cultivars sampled, sequence accession codes (BioProject number PRJNA796936), and original isolate references.

After grinding in liquid nitrogen, a Spectrum Plant Total RNA Kit (Sigma-Aldrich, Sydney, NSW, Australia) was employed to extract total RNA from 20 mg/sample (desiccated) or 50 mg/sample (fresh) leaf material. At the Australian Genome Research Facility (AGRF, Perth, WA, Australia), these extracts were assessed using a Nanodrop Spectrophotometer (Thermo Fisher Scientific Ltd., Melbourne, Victoria, Australia) before library preparation, barcoding (24 samples per lane), and 100 bp single-end sequencing on an Illumina HiSeq2000. In the UK, the Angua pipeline of Fowkes et al. [40] was used to analyze the paired reads as described by Jones et al. [16]. Complete or incomplete HPWMoV genome sequences were generated for each of these five samples (Table 1).

### 2.2. Sequence Analysis

The HPWMoV sequences were edited [41] to extract their main ORFs, and these were aligned using MAFFT [42]. RDP5.5 [43] was used to test for recombination using all its methods with default parameters [43,44,45,46,47,48,49,50,51,52]. Sequences with phylogenetic anomalies detected by five or more methods and with an average <10^−5^ chance probability were analyzed separately. The best substitution models for calculations involving the nt and encoded aa sequences were determined using MEGA11 [53] and found to be T92 + G + I [54] for nts and LG + G + I [55] for aa sequences. Phylogenetic trees were calculated by the neighbor-joining option in ClustalX [56] and by maximum likelihood (ML) using PhyML [57] with SH support statistics [58].

The ORFs of RNAs 1–4 from the CoPhil L1 isolate, the Nebraska “Genbank Reference Sequence” isolate, the West Australian Ku-12 isolate, and the Ohio isolate HPVWMoV-NW2 were each used as query sequences in BLASTX searches to identify the most similar 100 proteins in the Genbank protein database. The protein sequences for each RNA were pooled, and both duplicates and incomplete sequences were removed. The remaining 50–100 sequences for each RNA were aligned using MAFFT [42] and used to calculate ML phylogenies.

The RNA1 nts phylogeny was calculated from 12 out of 13 nt sequences downloaded, RNA2 from 15 of 17, RNA3 from 22 of 38, RNA4 from 18 of 53, RNA5 from all 14, RNA6 from 18 of 19, RNA7 from all 15 and RNA8 from all 16; those omitted were either incomplete or clearly mislabeled. The initial phylogenies of the RNA1 protein aa’s of different emaravirus species were calculated from 54 sequences selected from 113 downloaded, RNA2 aa’s from 63 of 112 downloaded, RNA3 aa’s from 157 of 168 downloaded, and RNA4 aa’s from 34 of 35 downloaded. Thus, a total of 130 out of 180 sequences for the RNA nt phylogenies, and 308 out of 428 for the protein aa phylogenies were used.

## 3. Results

### 3.1. Sample Origins

**Figure 1:**
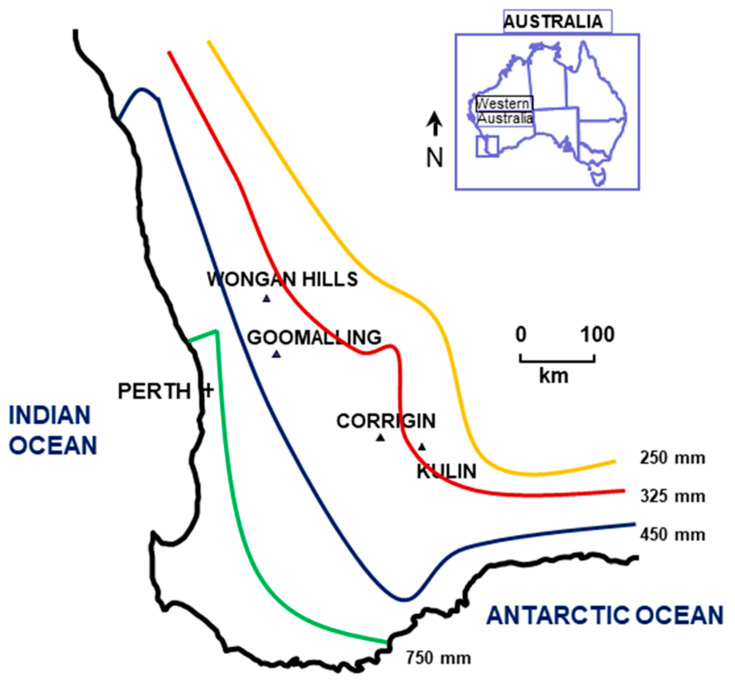
Collection sites in the south-west Australian grain belt where HPWMoV-infected wheat samples were obtained. The grain belt is subdivided into three zones with 250–325 (low), 325–450 (medium), and 450–750 (high) mm of rainfall per year.

Mace and Yitpi were the wheat cultivars from which the sequenced samples were obtained (Table 1). The HPWMoV sequences came from four WA locations (Corrigin, Goomalling, Kulin, and Wongan Hills), which were in the southern or central regions of the south-west Australian grain belt, occurring in their low or medium rainfall zones (Figure 1).

### 3.2. Recombination

Eight of the 31 RNA3 ORF sequences were found to be recombinants. Five of these (OM302271, OM302278, OM302287, OM302296 and OM302303) were among the 10 sequences from Australia, and three (MN315261, MN315262, MW990205) from among the 21 sequences from North America; all the others (KJ939625, KJ939626, KT970501, KT988862, KT988863, KT988871, KT988872, KT988881, KT988882, KT988889, KT995102, MN250339, MN250347, MT762120, MT762121, MT762122, MW990204 and NC_029550 from North America, and OM302270, OM302277, OM302286, OM302295 and OM302302 from Australia) were non recombinant (n-rec). The three recombinant sequences from North America came from isolates from Idaho (MN315261), Michigan (MN315262), and Kansas (MW990205), whereas the n-rec sequences came from isolates from Colorado (three), Ohio (nine), and three from unspecified sites in the USA. Tatineni et al. [35] reported “unusual heterogeneity in the nucleocapsid protein” of HPWMoV, namely the protein encoded by RNA3, and they distinguished two variants of the gene, RNA3A and RNA3B. Out of the 15 RNA3s currently identified as RNA3A or RNA3B, our analyses found that all eight RNA3A sequences are n-rec, whereas six of the seven RNA3B sequences are recombinant.

Seven of the eight recombinant RNA3 sequences had the same two closest “parental” sequences. The major “parent” was closest to the RNA3 of the type Nebraska isolate (NC_029550), and the minor “parent” was the “Nebraska-like” Ohio isolate (MN250339), involving the region from around nts 173 to 583 and detected by six methods with the probability that it occurred by chance ranging from a mean of 10^−5.5^ to 10^−7.2^. The other recombinant (MN315261) was an isolate from Idaho; its closest major “parent” was OM302295 from Australia, and its minor parent nts (248 to 456), were detected by six methods with the probability that it occurred by chance of <10^−9.0^.

### 3.3. Phylogeny

**Figure 2:**
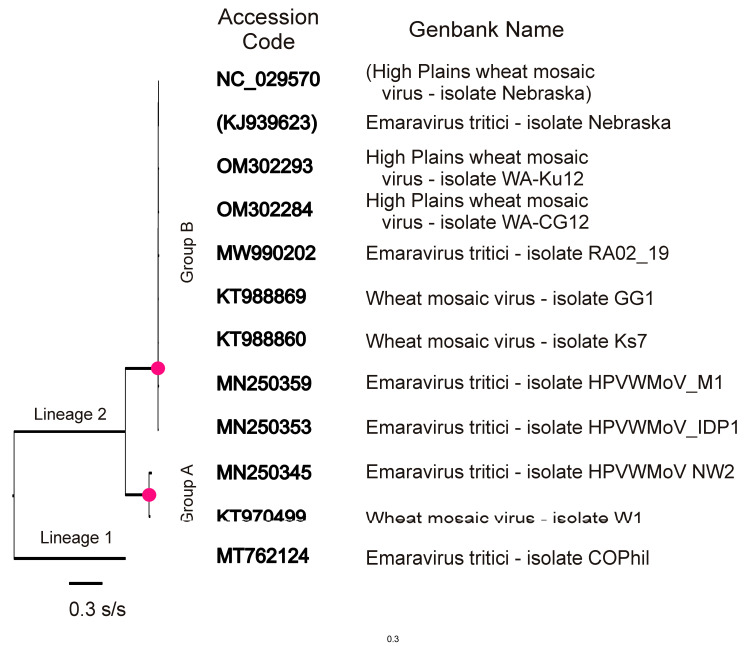
The ML phylogeny of the non-recombinant nucleotide sequences of 12 HPWMoV RNA1 ORFs. The geographical origins of these 12 sequenced isolates were: Western Australia (OM302293, OM302284), Nebraska (NC_029570, KJ939623), Kansas (MW99202, KT988860), Idaho (MN250353), Michigan (MN250359), Ohio (KT970499, KT988869, MN250345), and Colorado (MT762124). The Genbank record of the Nebraska isolate (KJ939623) uses the latinized binomial species name, *Emaravirus tritici*, now preferred by the International Committee on Taxonomy of Viruses. However, the Genbank Reference Sequence, NC_029570, is of the same record but, confusingly, uses the original vernacular name given to the virus.

The complete n-rec ORFs for each RNA segment were used to generate ML phylogenies. Figure 2 shows the RNA1 nt sequences, which, as mentioned above, encode the RdRp. All the other ORF sets gave similar phylogenies in that they also had two clear basal lineages, L1 and L2. L1 was of ORFs from a single isolate named “*Emaravirus tritici* COPhil,” and L2 was of all the other 11–20 ORF sequences (mean 14.75) of each HPWMoV gene. The L2 lineage is subdivided into two clusters, A and B. Cluster L2A consisted of two to six ORFs from wheat isolates all collected in Ohio; in Figure 2, they consisted of MN250345 and KT970499. Cluster L2B was composed of ORFs from 1–5 isolates collected from wheat, barley, or maize in Idaho, Kansas, Michigan, Nebraska, and Ohio in the GPR, and from wheat in WA in Australia. Five of the L2 ORFs were stated in Genbank to be from the same CoPhil isolate as the L1 ORFs. Four of these aberrant L2 ORFs (MTY762113, MT762115, MT762118, MT762120) were from RNAs 6, 5, 4 and 3, respectively, from one isolate with sequences found by BLAST analysis to be close to those of a group of L2A Ohio isolates, and the other, an RNA5 sequence (MT762115), was closest to the Nebraska type L2B sequence (NC_029552) and its source sequence (KJ939628).

**Figure 3:**
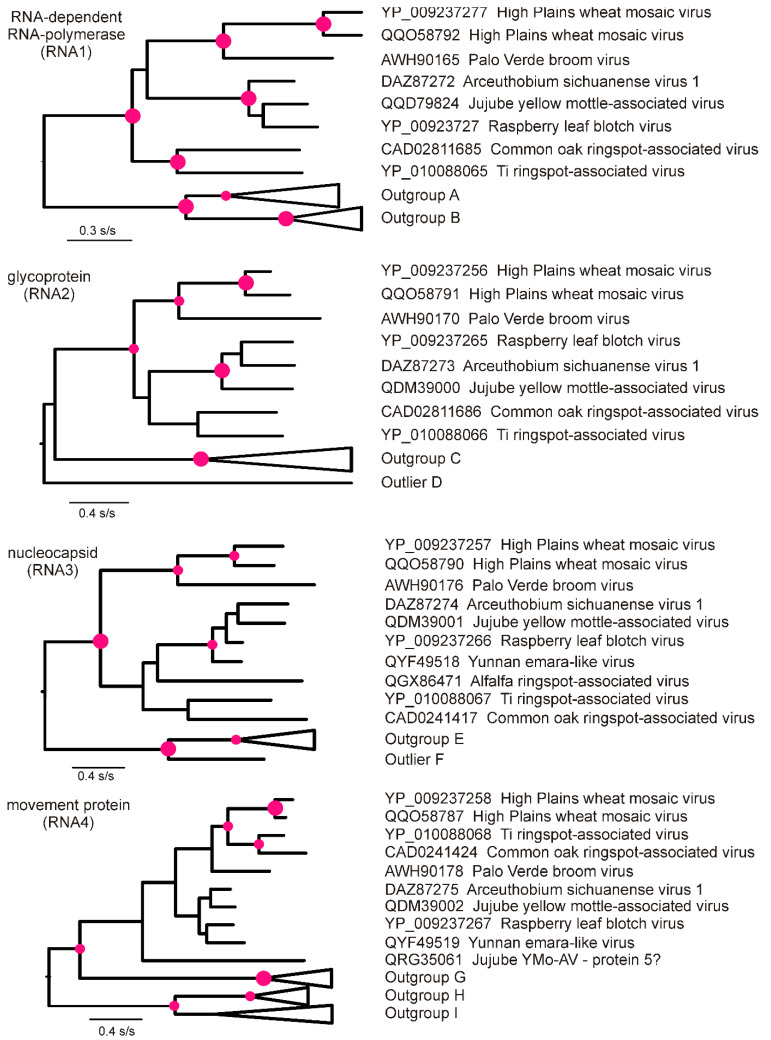
Phylogenies calculated from the amino acid sequences of the RNA-dependent RNA polymerase, glycoprotein, nucleocapsid, and movement proteins of four isolates of HPWMoV (only lineages 1 and 2 shown) and representatives of the closest proteins identified by a BLASTX search of Genbank—for details, see the text.

To check the more distant relationships of HPWMoV, the ORFs of the RNA1–4 genes from four representative HPWMoV isolates were used as query sequences in BLASTX searches of the GenbBank protein database to identify the closest non-HPWMoV proteins; the proteins encoded by RNAs 5–8 were not checked as these have not been identified for all emaraviruses. The phylogenies generated from these sequences (Figure 3) show that the RdRps, the glycoproteins, and the NCs of HPWMoV were closest to those of Palo Verde broom virus, and next closest to those of a cluster comprised of Arceuthobium sichuanense virus 1, Jujube yellow mottle-associated virus, raspberry leaf blotch virus, common oak ringspot-associated virus, and Ti ringspot-associated virus, along with, for the NC, Yunnan emara-like virus and alfalfa ringspot-associated virus. The phylogeny of the movement proteins was significantly different and showed the movement protein of HPWMoV to be closest to those of Ti ringspot-associated and common oak ringspot-associated viruses with 95% SH statistical support, indicating that reassortment was involved at some stage of the evolution of these viruses.

Large red discs on nodes with SH statistical support of 1.0 and smaller discs with 0.95 SH support. Isolates forming the HPWMoV-containing basal lineage are shown, but those of the other basal lineages/outliers are “collapsed” and consist of: Outgroup A: QAR18002, *Pistacia emaravirus*; QOI17315, Maple mottle-associated virus; Outgroup B: UQV97405, Peuraria montana associated virus; QTZ21230, and YP_010088071, Actinidia emaravirus; Outgroup C: YP_009237272, Emaravirus fici; YP_010088072, YP_010088072. Emaravirus kiwii, UQV97406 Pueraria lobata-associated emaravirus, CAG9003604; Ash shoestring-associated emaravirus, QAR18003 Pistacia emaravirus, YP_004327590 Emaravirus rosae, QOI17316 Maple mottle-associated virus, ANQ90730 Emaravirus cajani, YP_009507926 Actinidia chlorotic ringspot-associated virus; YP_009508087 Emaravirus cercidis; QIN85946 Lilac chlorotic ringspot-associated virus; Outlier D: QTW92691 Emaravirus camelliae; Outgroup E: AWS21342 Emaravirus fici; AXI82314 Emaravirus rosae; CAA0079646 Aspen mosaic associated virus, QBM15198 blackberry leaf mottle associated virus, UQV97407 Pueraria lobata associated emaravirus, YP_009268864 Emaravirus toordali; Outlier F: YP_009508085 Emaravirus cercidis; Outgroup G: QGX73506 Emaravirus camelliae, BCO17111 Japanese star anise ringspot-associated virus; Outgroup H: YP_009508084 Emaravirus cercidis, QIN85948 Lilac chlorotic ringspot-associated virus; Outgroup I: CAA0079685 Aspen mosaic-associated virus; QVW29594 Grapevine emaravirus A, YP_009237264; Emaravirus cajani movement protein sequence QRG35061 (along with QQD79828, QQD79841, and QDM39003) are mislabeled as RNA5 proteins in Genbank.

## 4. Discussion

Here we report a study of the relationships between HPWMoV isolates from Australia with other isolates from this same species, sometimes recorded under one of three (High Plains virus, wheat mosaic virus, or *Emaravirus tritici*) of its four other names. Comparisons with previously published analyses may be different or incomplete as the number of emaravirus sequences in Genbank is increasing, and, for this study, we used the complete ORF nt sequences of all HPWMoV genes. By contrast, Pozhylov et al. [12] used only partial gene sequences of the NC gene, although from more isolates, and did not identify and remove recombinant sequences of that gene. Furthermore, Tatineni et al. [35] and Stewart [37] calculated phylogenies from the aa sequences of HPWMoV genes, and so their phylogenies may be less discriminatory than ours in comparisons of closely related isolates but more discriminatory in more distant comparisons. Our analyses found that the HPWMoV ORFs of all genes grouped into two lineages, L1 and L2, confirming the findings of Stewart [37]. L1 consisted of a single Colorado isolate submitted to Genbank in 2021 without further details. L2 formed two clusters, named A and B by Pozhylov et al. [12]. The smaller L2A cluster was mostly of wheat isolates from Ohio, and the larger L2B cluster came from wheat, barley, and maize from several mid-west states of the USA and from wheat in Australia. The combined inference from all four different phylogenies ([12,35,37] and this paper) is that the HPWMoV found in Australia probably entered in plant material or seed from the USA, not from Europe or South America. This is what occurred when WSMV, which is known to be seed-borne in wheat, appeared in Australia [13,15,16], so the HPWMoV situation seems reminiscent of the WSMV situation. As mentioned in the Introduction (Section 1), HPWMoV is seed-borne in sweet corn [18,25,26], but such transmission, although suspected, is yet to be published for wheat (See Section 1, end of first paragraph).

The evidence of recombination is clear, but its interpretation will be more certain when the sequences of more closely related HPWMoV isolates are known. Seven of the recombinants are probably the progeny of a single recombination event that occurred somewhere in North America before its progeny were taken to Australia. By contrast, the Idaho recombinant (MN315261) is more likely to have had a major North American parent, of which the Australian OM302295 is merely the closest known progeny.

One of the USA states supplying the cluster L2B isolates was Idaho (MN250353), which is listed in Bicon (Australian Biosecurity Import Conditions; https://bicon.agriculture.gov.au/BiconWeb4.0/ImportConditions/Questions/EvaluateCase?elementID=0000068254&elementVersionID=294; accessed on 2 November 2022) as a permitted source of certified maize seed for entry to Australia. As mentioned above, HPWMoV has been found to be transmitted in sweet corn seed [18,25,26], but not yet in wheat seed. As sweet corn and maize are grown in separate areas of Australia from wheat, it is more likely that HPWMoV came to Australia in the seed of wheat breeding material, as occurred previously with WSMV [16,59]. HPWMoV possibly resembles WSMV in having its origin in the original wheat domestication center in the Middle East [16]. However, evidence for this is lacking currently due to the absence of any HPWMoV sequences from that region of the world. The year WSMV arrived in Australia was calculated to be only 2–3 years before it was first reported in Australia in 2002 [16]. HPWMoV was first found in Australia in 2003 [22]. Therefore, it likely arrived at the same time as WSMV.

The relationship between HPWMoV and other emaraviruses was investigated using the aa sequences of emaravirus genes identified by BLASTX searches of the Genbank database. Emaraviruses have been found in a wide variety of plant species, both monocotyledonous and dicotyledonous, annuals and perennials. Most of these records are from the northern hemisphere. The phylogenies for the RdRp, the glycoprotein, and the NC were closely similar (Figure 3). They placed HPWMoV closest to the Palo Verde broom virus [60]. However, the movement protein phylogeny placed HPWMoV closer to two other emaraviruses, Ti (Cordyline) ringspot-associated virus [61] and common oak ringspot-associated virus [62]. This finding confirmed the relationships shown, in part, in Figure 5A–D of the paper by Olmedo-Velarde et al. [61]. Therefore, we conclude that emaravirus genetic diversity is produced not only by mutation but also by reassortment of gene segments and recombination within gene segments. Finding clear evidence of recombination in the RNA3 (NC) gene of HPWMoV was unexpected, as Boni et al. [63] concluded that “homologous recombination is very rare or absent in” the human influenza A virus and other viruses with negative-strand RNA genomes. It is noteworthy that the proportion of RNA3 recombinants was greater in the Australian isolates than those from elsewhere: 5/10 (50%) of isolates from Australia compared with only 3/21 (14%) of isolates from other world regions. They were probably the progeny of only two recombination events, which are more likely to have taken place in the GPR than in Australia, as this would have involved fewer transcontinental journeys.

HPWMoV epidemics in wheat depend not only on the ecology and behavior of its WCM vector, which requires high temperatures for its rapid population build-up, but also on the nearby presence of HPWMoV-infected host plants from which WCM can spread the virus to recently sown wheat crops [18]. In the GPR, wheat is often sown in the autumn, shortly after, or even coincident with, the time when the preceding crop is harvested. The HPWMoV-infected host plants from which WCM spreads it to newly sown wheat crops consist mainly of a “green bridge” of volunteer wheat plants surviving in between successive wheat crops or of as yet unharvested mature wheat, sweet corn, or maize crops from the previous annual sowing that overlap with newly planted wheat crops [18]. The potential role of HPWMoV seed transmission in wheat, sweet corn, or maize in its disease cycle appears not to have been considered for the situation where successive wheat crops do not overlap. This contrasts with the situation in parts of Australia with Mediterranean-type climates, where a protracted hot, dry summer period intervenes between wheat crop harvests in late spring and wheat plantings in late autumn. Under these circumstances for WSMV, seed-borne infection in wheat plays a critical role in the persistence of this virus between successive wheat crops. Seed-infected seedlings arising from volunteer wheat plants or from sowing contaminated wheat seed stocks act as primary infection foci for WCM to spread the virus within wheat crops [15]. Further research is needed to determine whether HPWMoV is seed-borne in wheat and whether seed transmission in wheat plays a similar role in its epidemiology.

WSMD is most damaging when co-infections with its causal viruses (WSMV, HPWMoV, and TriMV) occur (see Section 1). The spread of HPWMoV to countries in which it is currently absent but WSMV and its WCM vector are present is cause for concern for their wheat industries, necessitating consideration of biosecurity measures to prevent such introduction. This is especially true due to the projected increase in importance of WSMD worldwide as global warming magnifies losses from the current global epidemic. The same would also hold true for TriMV if it spread from the GPR to other world regions with significant wheat industries, particularly those where epidemics of the other two viruses already occur.

## Figures and Tables

**Table 1:** Virus-infected samples of wheat from south-west Australia that contained High Plains wheat mosaic virus: sample names, virus(es) present, origins, and sequence accession codes.

Sample (Isolate)	Virus(es) Found ^A^	Isolation Year	Location Sampled	Wheat Cultivar	HPWoMV Sequences	Sequences Obtained	HPWoMV Accession No.	Original Sample/Isolate Reference
BCWS5 (WA-Ku12A)	HPWMoV + WSMV	2012	Kulin	cv. Mace	Partial	RNA3A, RNA3B, RNA6	OM302302-4	Coutts et al. [23]
BCHPV1 (WA-Ku12)	HPWMoV + WSMV	2012	Kulin	cv. Mace	Complete coding sequences	RNA1, RNA2, RNA3A, RNA3B, RNA4, RNA5, RNA6, RNA7, RNA8	OM302293-301	Coutts et al. [23]
BCHPV2 (WA-CG12)	HPWMoV	2012	Corrigin	cv. Yitpi	Complete	RNA1, RNA2, RNA3A, RNA3B, RNA4, RNA5, RNA6, RNA7, RNA8	OM302284-92	Coutts et al. [23]
HP1G (WA-GM-13)	HPWMoV + WSMV	2013	Goomalling	cv. Mace	Partial	RNA2, RNA3A, RNA3B, RNA4, RNA5, RNA6, RNA7, RNA8	OM302276-83	Jones et al. [16]
HP2W (WA-WH-13)	HPWMoV + WSMV	2013	Wongan Hills	cv. Mace	Partial	RNA3A, RNA3B, RNA4, RNA6, RNA7, RNA8	OM302270-75	Jones et al. [16]

**^A^** Virus acronyms: High Plains wheat mosaic virus (HPWMoV), wheat streak mosaic virus (WSMV). WSMV and HPWMoV presence in samples BCWS5, BCHPV1, HP1G and HP2W was reported previously [16].

## Data Availability

The data that support the findings of this study are openly available in GenBank at https://www.ncbi.nlm.nih.gov/genbank (our data was added to Genbank on 21 September 2021). Individual virus isolate sequence data are available in NCBI Genbank under the accession codes listed in Table 1. All sequence data derived from the samples reported here can be found in the NCBI short read archive, BioProject PRJNA796936.
